# Upregulation of chemokine CXCL10 enhances chronic pulmonary inflammation in tree shrew collagen-induced arthritis

**DOI:** 10.1038/s41598-018-28404-y

**Published:** 2018-07-03

**Authors:** Bo Gao, Jie Lin, Zongmin Jiang, Zhongshan Yang, Haijing Yu, Lei Ding, Min Yu, Qinhua Cui, Neil Dunavin, Ming Zhang, Meizhang Li

**Affiliations:** 1 Lab of Biochemistry & Molecular Biology, School of Life Sciences, Yunnan University, Kunming, Yunnan 650091 China; 2 Key Lab of Molecular Cancer Biology, Yunnan Education Department, Kunming, Yunnan 650091 China; 3 Lab of Monoclonal Antibody Engineering &Technology, School of Life Sciences, Yunnan University, Kunming, Yunnan 650091 China; 4 Lab of Pathogen Biology and Immunology, Yunnan Traditional Chinese Medicine University, Kunming, Yunnan 650500 China; 5 Division of Hematologic Malignancies and Cellular Therapeutics, Kansas University Medical Center, KS, 66205 USA; 6 Yunnan Key Laboratory of Stem Cell and Regenerative Medicine, Institute of Molecular and Clinical Medicine, Kunming Medical University, Kunming, Yunnan 650500 China

## Abstract

Chronic pulmonary inflammation (CPI) gives rise to serious lung injuries in rheumatoid arthritis (RA) patients. However, the molecular mechanism underlying the pathogenesis of RA-associated CPI remains little understood. Here we established a novel tree shrew-based collagen-induced arthritis (TsCIA) model to study RA-associated CPI. Our results showed that typical CPI but not fibrosis developed pathologically in the TsCIA model. Furthermore, abnormal up-regulation of pulmonary chemokine CXCL10 was directly associated with lung damage. Specific blockage of CXCR3 (a CXCL10 receptor) significantly decreased the severity of CPI by decreasing the recruitment of inflammatory cells. Therefore, CXCL10 is proposed as a key player responsible for the development of TsCIA-associated CPI. Our findings also suggest that CXCR3 could be developed as a potential diagnosis biomarker for RA-associated CPI.

## Introduction

Rheumatoid arthritis (RA) is a chronic inflammatory autoimmune disorder with long-term inflammation of joint synovia and progressive destruction of cartilage and bone^1,2^. The clinical development of RA is directly related to the abnormal infiltration of inflammatory cells such as T and B lymphocytes, neutrophils, monocytes, and macrophages into synovial tissues, joints, and other organs^3–6^. These infiltrated inflammatory cells mainly rely on chemokines and their cognate receptors to functionally mediate their migration from circulation to the organs^5,6^. In addition to joint inflammation, interstitial lung diseases (ILDs) have recently gained attention because serious lung damage also affects RA patient life quality, and may even cause their mortality^7,8^. Clinically, 19–56% and 25–30% of RA patients develop ILDs or air-way diseases, respectively^9,10^. RA-associated ILDs include idiopathic interstitial pneumonia and idiopathic pulmonary fibrosis^11^. RA patients with ILDs are frequently diagnosed with scarred and thickened alveoli due to robust tissue repair after lung injury^12,13^. Although infiltrated inflammatory cells are reportedly responsible for both lung injury and repair in RA patients^14,15^, whether and how chemokines mediate the infiltration of inflammatory cells in RA-associated lung diseases remain poorly understood.

Tree shrews (*Tupaia belangeri chinensis*) are native species to Southern Asia, Southeast Asia, and Southwest China^16^. These unique animals display functional maps and neural connections in the cortex as complex as those of humans^17^ and exhibit the same brain trait distribution patterns as those of primates^18^. Whole genome phylogenetic analysis has shown that tree shrews are evolutionarily closer to primates than to rodents^16,19^. Consistent with this, we previously determined that the CXCL12 chemokine is structurally- and functionally-conserved in tree shrews and primates^20^. Thus, tree shrews are increasingly considered to be suitable animal models for complex human diseases and have been successfully applied in many studies related to virus infection and tumorigenesis^21–23^. Here, for the first time, we established a tree shrew-based collagen-induced arthritis (TsCIA) model for studying RA-associated ILDs, with inflammatory reactions in both joints and lungs further characterized. The relevance of TsCIA-associated ILDs with chemokines was also investigated.

In both RA patients and collagen-induced arthritis (CIA) animal models, interferon γ (IFN-γ) inducible chemokine IP10 (CXCL10) regulates the pathological development of joint inflammation^24–34^. CXCL10 is also involved in the pulmonary pathogenesis of different pathologies and has been suggested as a biomarker for ILDs^35,36^. In the adoptive transfer of both alloreactive and systemic lupus erythematosus models, CXCL10 amplifies Th1-mediated pulmonary inflammation^37,38^. Furthermore, transient expression of CXCL10 facilitates the recruitment of neutrophils and mononuclear cells to the lungs in adenovirus-induced pulmonary inflammation models^39^. Genetic disruption of the CXCR3 (a CXCL10 receptor) gene reduces leukocyte recruitment and decreases lung inflammation in both idiopathic pneumonia syndrome and cigarette smoke models^40,41^. In addition, CXCL10 has been suggested as an anti-fibrotic gene in both idiopathic pulmonary fibrosis and bleomycin-induced pulmonary fibrosis^42–44^. However, the detailed roles of CXCL10 in RA-related ILDs remain to be determined.

## Results

### Establishment of TsCIA model

**Figure 1:**
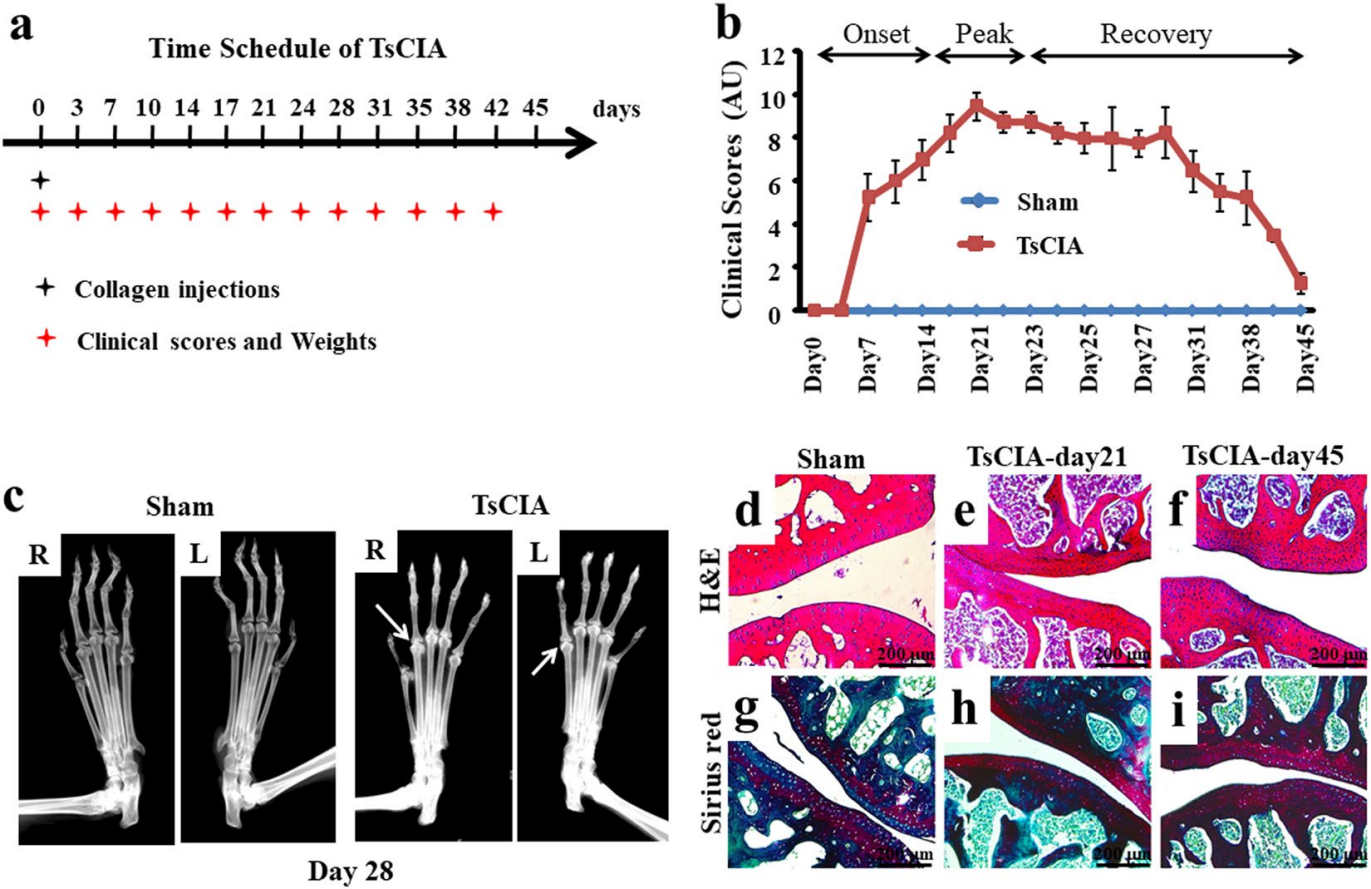
Establishment of tree shrew collagen-induced arthritis (TsCIA). (**a**) Time schedule for the establishment of TsCIA. (**b**) Clinical scores of the control and TsCIA groups (n = 20 tree shrews per group). AU indicates arbitrary unit. (**c**) Representative X-ray images from control and TsCIA forelimbs at day 28. R, right and L, left. Arrows indicate damaged joints in TsCIA. (**d–i**) Histochemical analysis of joint cross-sections from the same tree shrews shown at day 21 and day 45. H&E staining shows the endogenous chondrocytes in (**d–f)**. Sirius red and solid green staining show cartilage damage (arrows) in (**g–i**). Scale bar, 200 μm.

To establish the TsCIA model, tree shrews were immunized by subcutaneous injection of bovine type II collagen, with a standard clinical scoring system then used to evaluate the severity of joint symptoms (Fig. 1a). As seen in Fig. 1b, the severity of joint inflammation worsened over two weeks (days 7–21) before reaching a maximal peak stage at day 21. After day 23, joints showed remission and entered a long-term recovery stage (days 23–45). No significant weight difference was observed between the TsCIA and control tree shrews during the period of collagen immunization (Supplementary Fig. S1). To verify the joint symptoms in TsCIA animals, we examined forelimb joint damage by X-ray analysis. At day 28, multiple pathological changes such as soft tissue swelling, joint space narrowing, and marginal erosions were observed (Fig. 1c, arrows). To confirm these observations, we examined the histological changes in joint tissues sectioned from TsCIA and control animals (Fig. 1d–i). At day 21, H&E staining demonstrated denser and abnormal lymphoid infiltrates within the tissues cross-sectioned from bone joints and the gap between bone joints also became narrower in TsCIA animals (Fig. 1e) compared with the control tree shrews (Fig. 1d). In addition, cartilage damage was frequently observed in the TsCIA joints (Fig. 1e). It is worth mentioning that high-density chondrocytes predominantly appeared around the damaged areas (Fig. 1e). Although cartilage damage and chondrocyte proliferation remained, the gap between the joints returned to normal in TsCIA animals at day 45 after overall improvement of joint symptoms (Fig. 1f). Sirius red staining confirmed that collagen fibers were markedly increased in the TsCIA cartilage due to tissue damage with the development of RA (Fig. 1g–i, arrow). Therefore, both clinical scoring and histological analysis demonstrated successful establishment of the TsCIA model.

### Pulmonary inflammation in TsCIA

**Figure 2:**
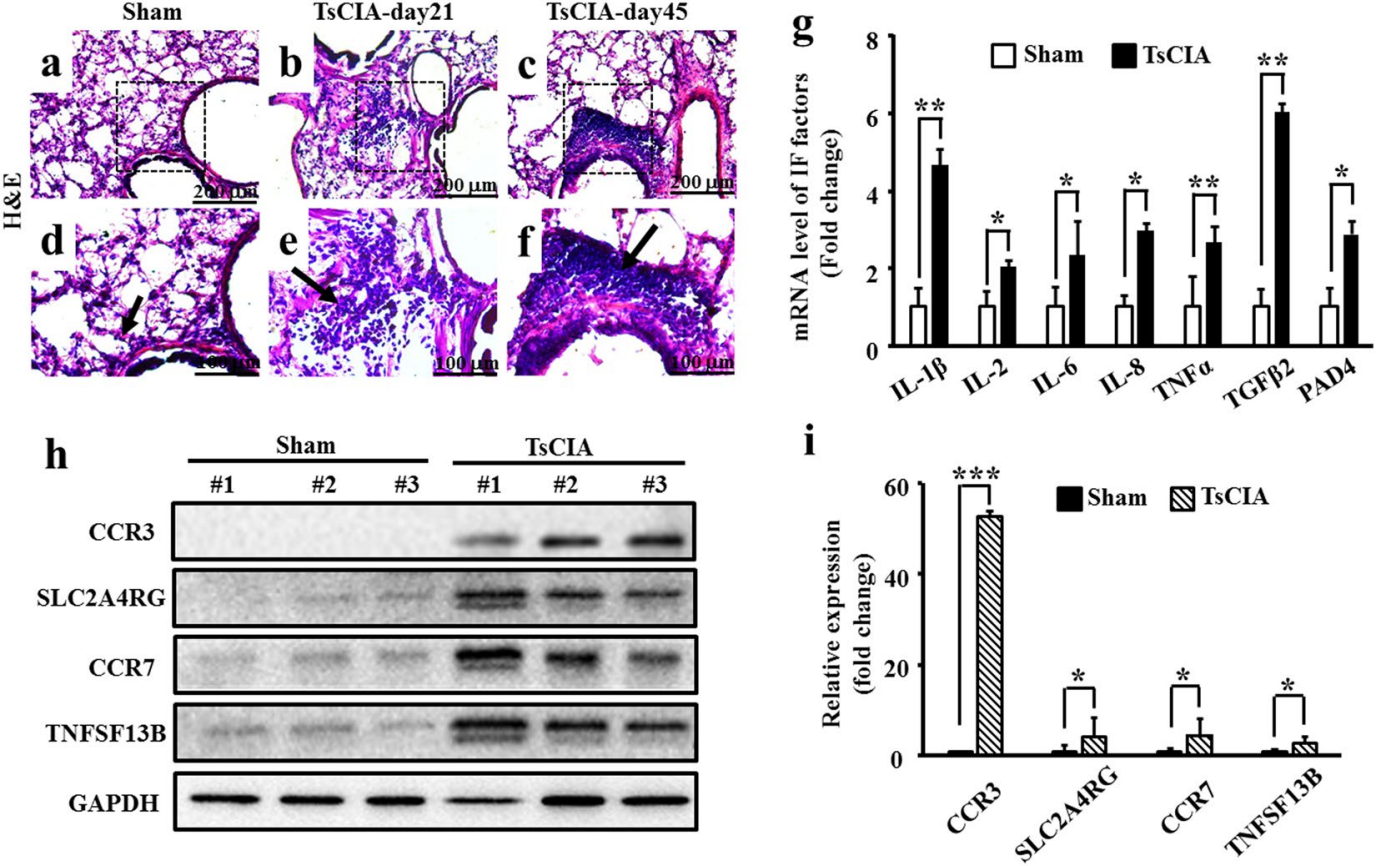
Pulmonary inflammation in TsCIA. (**a–f**) The PBMCs were detected in TsCIA lungs at day 21 and day 45. Arrows indicate infiltrated PBMCs. Scale bar: 200 μm (**a–c**); 100 μm (**d–f**). (**g)** Quantification of mRNA levels of lung inflammatory factors (n = 4 tree shrews per group, *p < 0.05, **p < 0.01. Error bars indicate SEM). (**h**) Protein expressions of inflammatory factors in the TsCIA lungs detected by Western blot analysis. (**i**) Semi-quantification of the relative expression levels of CCR3, SLC2A4RG, CCR7, and TNFSF13B proteins, normalized to GAPDH compared with the control tree shrews from F (n = 4 tree shrews per group, *p < 0.05, ***p < 0.001. Error bars indicate SEM).

We next characterized pulmonary manifestations and inflammation in TsCIA. We conducted H&E staining to examine for inflammatory cell infiltrates in lung tissue. While a small number of inflammatory cells were found in the control lungs (Fig. 2a and d, arrow), many diffused lymphoid infiltrates were observed in the TsCIA lung tissue at day 21 (Fig. 2b and e, arrow). In particular, we observed lymphoid infiltrates predominantly within the perivascular and peribronchiolar regions at day 45 (Fig. 2c and f, arrow). Further cell typing showed that the pulmonary inflammatory infiltrates were mainly lymphocytes and monocytes, with a small number of eosinophils and neutrophils also found in the lungs of TsCIA animals (Supplementary Fig. S2). These observations suggest that pulmonary inflammation occurred due to the systematic activation of the immune response in the TsCIA model. To confirm these results, we examined the expressions of some pro-inflammatory factors in TsCIA lung tissue (Fig. 2g–i). Real-time PCR showed that the mRNA levels of IL-1β, IL-2, IL-6, IL-8, TNFα, TGFβ2, and PAD4 were significantly up-regulated in the TsCIA lung tissue compared with those in the control (Fig. 2g). Western blot analysis also showed that the protein levels of CCR3, SLC2A4RG, CCR7, and TNFSF13B were increased in the TsCIA lungs compared with those in the control (Fig. 2h and i). Therefore, histochemical and molecular evidence supported that both pneumonitis and arthritis developed in the TsCIA model. To determine whether TsCIA further developed fibrotic lung disease, we performed Masson staining to detect fibrosis in the TsCIA lung tissue. However, no obvious fibrosis was observed (Supplementary Fig. S3), suggesting that if pulmonary fibrosis occurs it is more likely a late manifestation of TsCIA-related ILD.

### CXCL10 expression and distribution in TsCIA lung tissue

**Figure 3:**
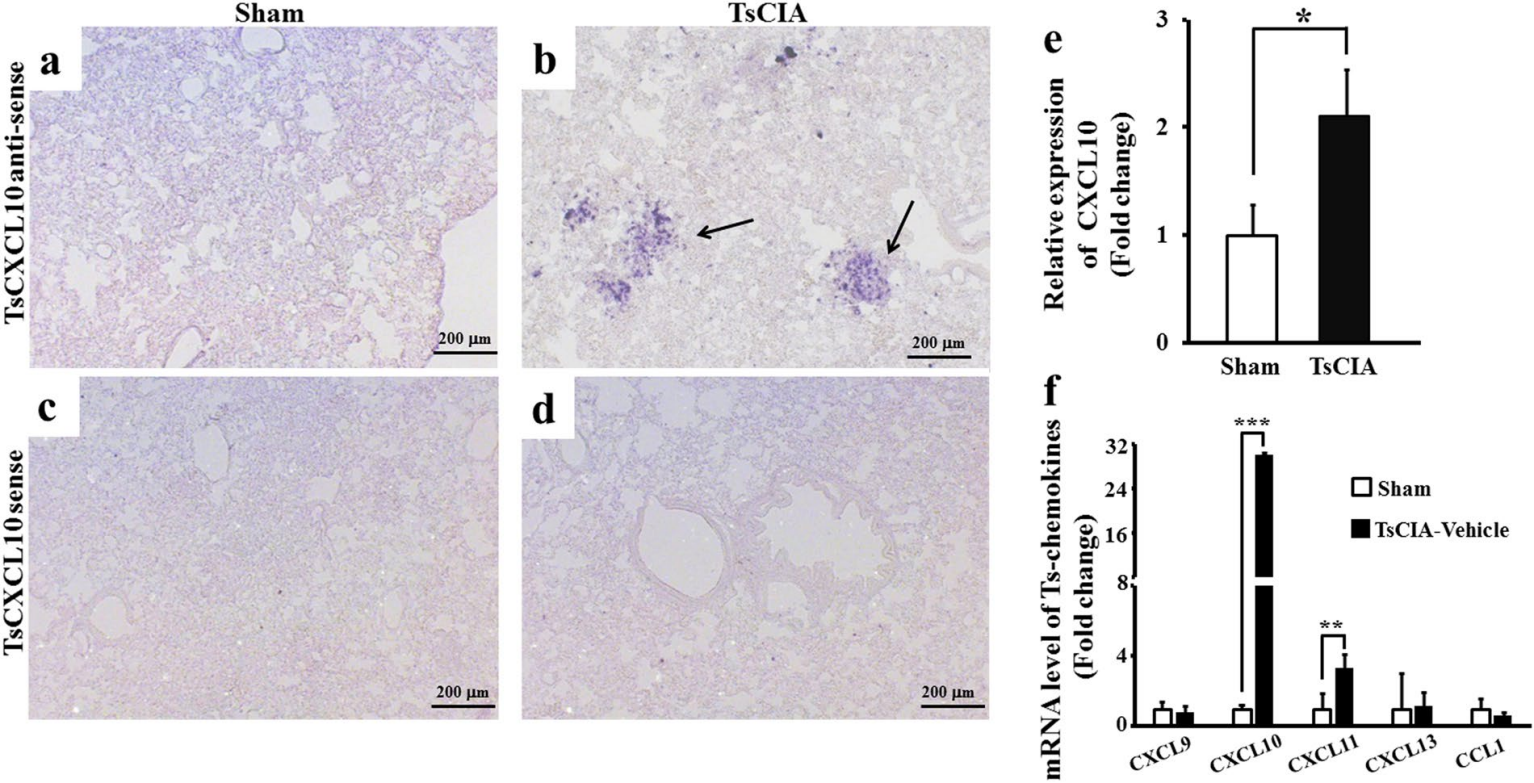
Up-regulation of CXCL10 in the lungs of TsCIA. (**a–d**) mRNA expression and distribution of CXCL10 were detected by *in situ* hybridization (ISH) in TsCIA lungs at day 45. Representative ISH signals are indicated by arrows. Hybridizations were performed by antisense (**a** and **b**) or sense probes (**c** and **d**), respectively. Control (**a** and **c**); TsCIA (**b** and **d**); Scale bar, 200 μm. (**e**) ISH signal area was analyzed by NIH ImageJ software (*p < 0.05. Error bars indicate SEM). (**f**) Quantification of mRNA levels of chemokines in control lungs and TsCIA lungs (n = 4 tree shrews per group, **p < 0.01, ***p < 0.001. Error bars indicate SEM).

To investigate the possible role of CXCL10 in TsCIA-associated pulmonary inflammation, the expression and distribution of CXCL10 mRNA in TsCIA lung tissue were examined by *in situ* hybridization (ISH). We found that CXCL10 mRNA levels were markedly increased in the TsCIA lungs compared with levels in the control lungs (Fig. 3a–d). We also examined the mRNA expression pattern of CXCL10 in TsCIA lung tissue. The ISH results revealed agglomerative and dispersive expression patterns, with agglomerative and dispersive cells both expressing CXCL10 mRNA. Agglomerative cells tended to cluster within a confined locality, whereas dispersive cells were scattered in the surrounding lung tissue (Fig. 3b, arrows). These two expression patterns were not observed in the control lung tissue (Fig. 3a). Thus, we inferred that CXCL10-expressing cells were infiltrating cells. To avoid nonspecific hybridization signals, sense probes for CXCL10 were used to hybridize series sections from the same lung tissue samples. No ISH signals were detected when CXCL10 sense probes were used (Fig. 3c and d). The relative expression of CXCL10 in TsCIA lungs exhibited a 2-fold increase compared with that in the control lungs (Fig. 3e). The ISH findings indicate that local up-regulation of CXCL10 coincided with lung injury in the TsCIA model.

Using real-time PCR, we further detected the mRNA levels of different chemokines in TsCIA-associated pulmonary inflammation. Results showed that both CXCL10 and CXCL11 mRNA levels were significantly increased in the TsCIA lungs compared with levels in the control lungs (Fig. 3f). Specifically, we observed a more than 20-fold increase in CXCL10 mRNA levels in the TsCIA lungs compared with levels in the control group. No significant differences were observed in CXCL9, CXCL13, or CCL1 mRNA levels between the two groups. Furthermore, there were no obvious changes in the protein levels of CXCL10 in peripheral blood serum between the TsCIA and control groups (Supplementary Fig. S4a), and no significant changes in the expression of CXCR3 mRNA in peripheral blood mononuclear cells (PBMCs) (Supplementary Fig. S4b) or lungs (Supplementary Fig. S5) at different times between the TsCIA and control groups. These findings strongly suggest that the localized secretion of chemokine CXCL10 to lung tissue coincided with pulmonary inflammation in the TsCIA model.

### Alleviation of TsCIA-induced arthritis and pulmonary inflammation by CXCR3 blockage

**Figure 4:**
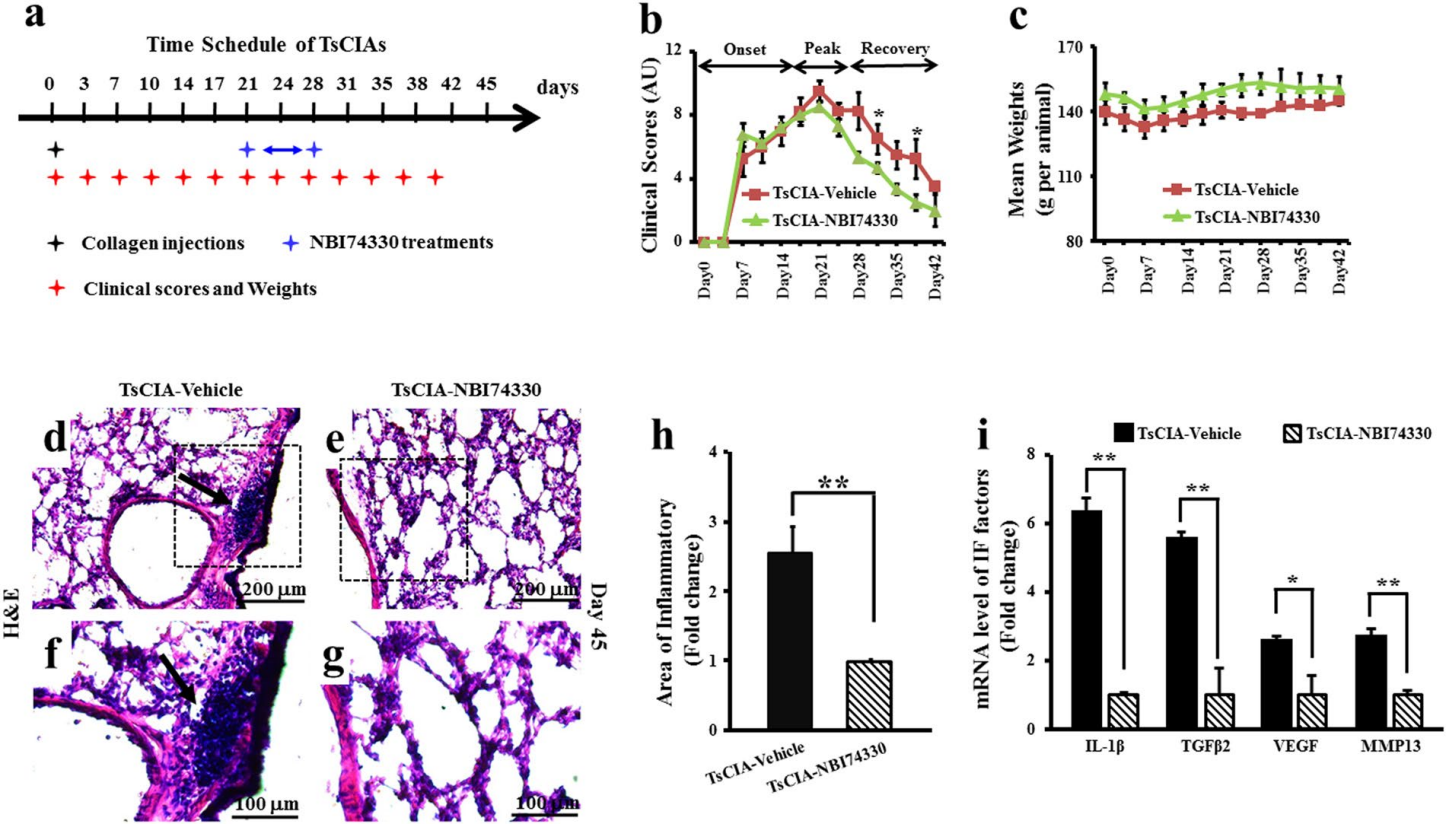
Recovery of TsCIA following *in vivo* treatment with NBI74330. (**a**) Time schedule for NBI74330 treatment in TsCIA. (**b**) Clinical scores of TsCIA treated with CXCR3-specific antagonist NBI74330 in (**a**) (n = 4 tree shrews per group, *p < 0.05). AU indicates arbitrary unit. (**c**) Average body weights. (**d–g**) H&E images of lung tissues in vehicle- or NBI74330-treated TsCIA at day 21. Infiltrated PBMCs are indicated by arrows. Scale bar: 200 μm (**d** and **e**) and 100 μm (**f** and **g**). (**h**) Infiltrated cell area was compared between TsCIA and TsCIA-NBI74330 by NIH ImageJ software (**p < 0.01. Error bars indicate SEM). (**i**) Quantification of mRNA levels of the inflammatory factors in TsCIA lungs and TsCIA-NBI74330 lungs (n = 4 tree shrews per group, *p < 0.05, **p < 0.01. Error bars indicate SEM).

The up-regulated expression of CXCL10 in TsCIA lungs raises the possibility that CXCL10-CXCR3 chemotaxis is necessary for pulmonary inflammation. To address this hypothesis, the CXCR3 receptor-specific antagonist NBI74330 was used to test whether CXCR3 blockage alleviated both joint and pulmonary inflammation in the TsCIA model. NBI74330 was intraperitoneally administered at a dose of 1 mg/kg on days 21–28 after collagen immunization (Fig. 4a). Clinical scores showed that NBI74330 administration significantly accelerated the recovery of joint inflammation in the TsCIA model compared with that of the vehicle-treated control group (Fig. 4b), and no significant weight difference was observed between the TsCIA-NBI74330 and TsCIA-vehicle tree shrews (Fig. 4c). These findings suggest that CXCL10-CXCR3 chemotaxis mediated joint inflammation in TsCIA.

**Figure 5:**
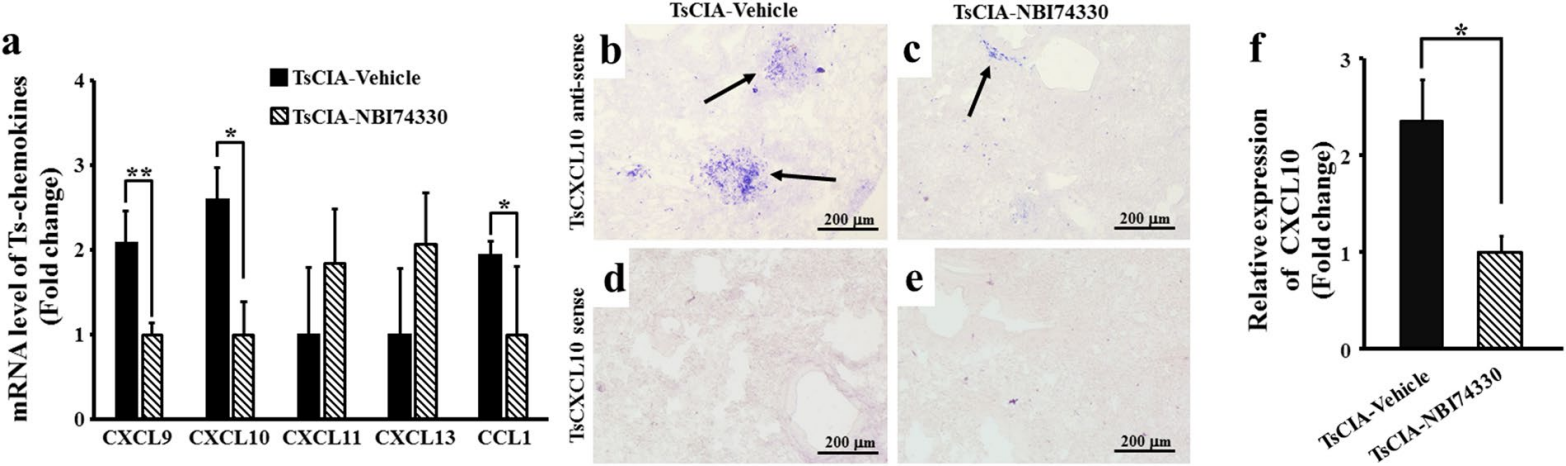
Expression of chemokines in TsCIA lungs. (**a**) Quantification of mRNA levels of chemokines in TsCIA lungs and TsCIA-NBI74330 lungs (n = 4 tree shrews per group, *p < 0.05, **p < 0.01. Error bars indicate SEM). (**b–e**) Representative ISH images show mRNA expression of CXCL10 after treatment with NBI74330. Arrows indicate positive ISH signals. Scale bar, 200 μm. (**f**) CXCL10 expression area was analyzed by NIH ImageJ software (*p < 0.05. Error bars indicate SEM).

We also examined the effects of NBI74330 on pulmonary inflammation in TsCIA. H&E staining demonstrated that the number of inflammatory infiltrates (Fig. 4d–g) and the area of inflammatory (Fig. 4h) were greatly decreased in the TsCIA-NBI74330 group compared with the TsCIA-vehicle group. The reduction in inflammatory infiltrates suggested that specifically targeting CXCR3 with NBI74330 reduced pulmonary inflammation. To verify this observation, real-time PCR was performed, which showed that the mRNA expressions of inflammatory factors (IL-1β, TGFβ2, VEGF, and MMP13) and chemokines (CXCL9, CXCL10, and CCL1) were significantly decreased between the TsCIA-NBI74330 and TsCIA-vehicle groups (Figs 4i and 5a). Results showed that the mRNA levels of these inflammatory factors were significantly down-regulated after treatment with the CXCR3 antagonist NBI74330.

The ISH experiment showed that CXCL10 mRNA expression was obviously decreased in the lung tissue of TsCIA-NBI74330 animals compared with that in TsCIA-vehicle animals (Fig. 5b–e). When CXCL10 sense probes were used, no ISH signals were detected for the two groups (Fig. 5d and e). There was a more than 2-fold increase in the expression of CXCL10 in the lungs between the TsCIA-NBI74330 and TsCIA-vehicle groups (Fig. 5f). These results indicated that the expression of CXCL10 in lung tissue was significantly reduced after NBI74330 treatment in the TsCIA animals compared with that in the TsCIA-vehicle group.

### CXCL10-induced transmigration of PBMCs

**Figure 6:**
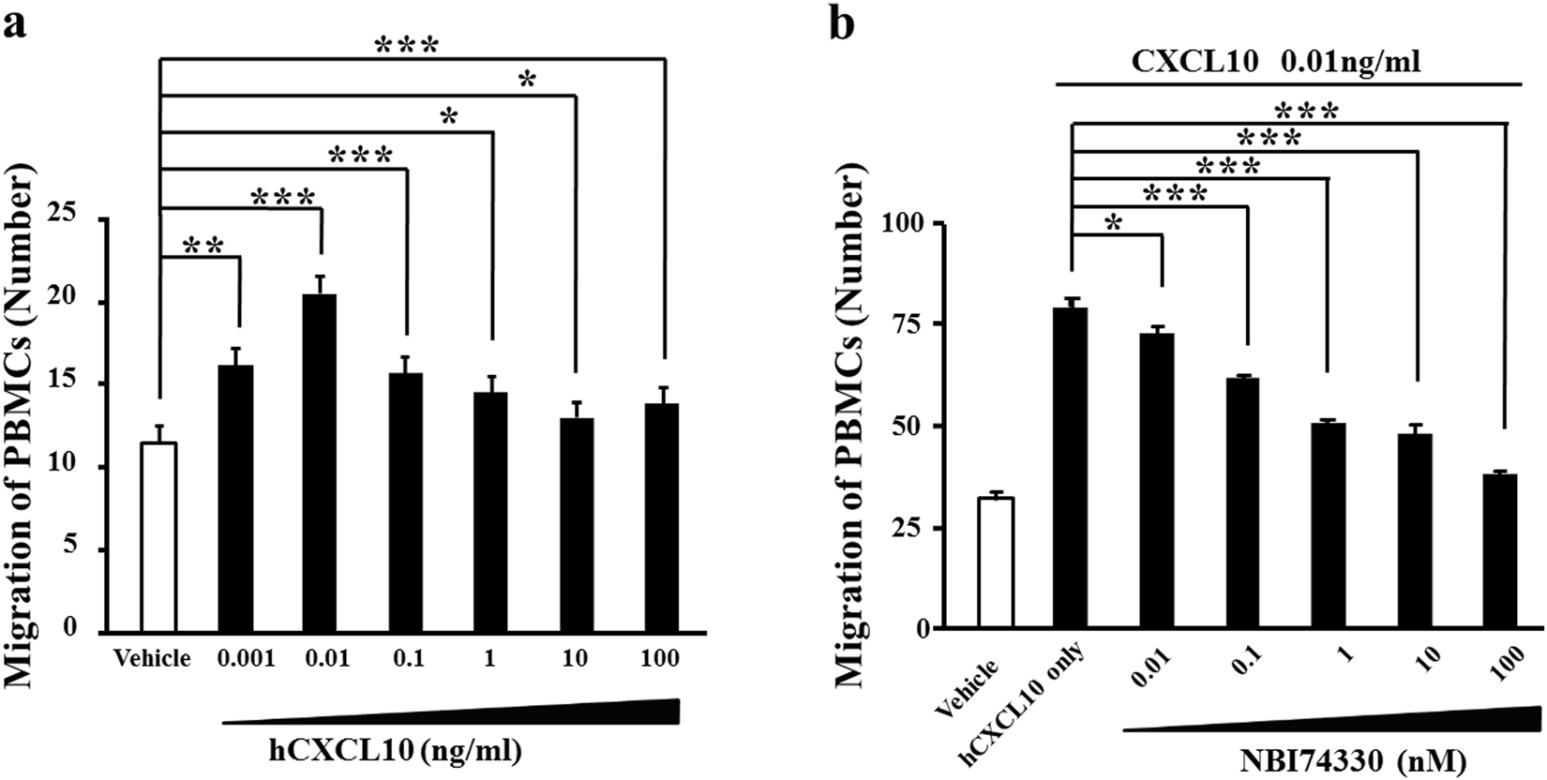
Migration of tree shrew’s PBMCs depended on CXCL10-CXCR3 function. (**a**) Quantification of *in vitro* transmigration of tree shrew’s PBMCs induced by recombinant human CXCL10 (n = 9 wells per group, *p < 0.05, **p < 0.01, ***p < 0.001). (**b**) Targeting CXCR3 by NBI74330 efficiently blocked hCXCL10-induced *in vitro* transmigration of tree shrew’s PBMCs (n = 9 wells per group, *p < 0.05, ***p < 0.001). Error bars indicate SEM.

We conducted *in vitro* experiments to detect whether CXCL10-CXCR3 chemotaxis is required for the recruitment of peripheral inflammatory cells in tree shrews. As shown in Fig. 6a, recombinant human CXCL10 (hCXCL10) at 0.001–100 ng/ml induced the *in vitro* transmigration of tree shrew’s PBMCs, with the most efficient dosage found at 0.01 ng/ml. The PBMCs transmigration induced by hCXCL10 at 0.01 ng/ml was effectively inhibited following treatment with 0.01–100 nM NBI74330 (Fig. 6b). The NBI74330 concentrations were safe and non-toxic against the treated PBMCs (Supplementary Fig. S6). These data indicate that the enhanced pulmonary inflammation in the TsCIA model may be through CXCL10-induced recruitment of peripheral inflammatory cells into lung tissue.

## Discussion

In this study, we successfully established a tree shrew-based CIA model for the first time. TsCIA developed typical joint pathologies similar to the clinical manifestations described in RA patients^1,2^ and rodent-based CIA^45^. Evidence from medical imaging and histochemical analysis further supported that TsCIA joints were damaged due to the abnormal activation and migration of peripheral inflammatory cells. More importantly, severe pulmonary inflammation was simultaneously observed in the TsCIA model. Similar to the joints in TsCIA, TsCIA-associated pulmonary inflammation was directly caused by the infiltration of inflammatory cells. Although the pathological development of TsCIA reached a maximal peak stage, pulmonary inflammation tended to develop over a long time (about four weeks). The process of pulmonary inflammation in the TsCIA model was different from that in rodents. Thus, the successful establishment of TsCIA provides a unique experimental model to explore RA-associated ILDs.

As important signaling proteins, secreted chemokines regulate joint inflammation through mediating the recruitment of peripheral inflammatory cells in RA patients and related models^5,6,24,30^. Up-regulated CXCL10 is observed in the synovial fluid of RA patients^24,25^. Consistently, CXCR3 is preferentially expressed in Th1 cells, B cells, dendritic cells, mast cells, and fibroblasts in the synovia^26–29^. Migration of Th1 cells from the blood to the synovial joints is dependent on the CXCL10 signaling pathway^24,30^. Inhibition of the interaction between CXCL10 and CXCR3 leads to diminished recruitment of Th1 type cells in RA patients^31–33^. Antibody-specific clearance of CXCL10 also significantly decreases the development of CIA^34^. Unfortunately, the roles of chemokines in RA-associated ILD remain poorly understood. In this study, we reported CXCL10 (or IP-10) as an important regulator for RA-associated ILDs. We found that the mRNA levels of CXCL10 were abnormally up-regulated in the TsCIA lungs due to tissue injury. Increased CXCL10 was responsible for the recruitment of inflammatory cells to the lungs, further enhancing pulmonary inflammation. Targeting CXCR3 with receptor-specific antagonist NBI74330 significantly decreased lung inflammation by inhibiting the migration of peripheral inflammatory cells. To the best of our knowledge, CXCL10 is the first identified chemokine shown to be involved in the pathological development of TsCIA-associated ILD. CXCL10 has been suggested as a biomarker for non-RA ILDs^35,36^, where it enhances both pulmonary inflammation and fibrosis through recruiting Th1 type or other inflammatory cells^37,38^. Our findings extend current knowledge on the essential roles of CXCL10 in autoimmune ILDs. In addition, we found that chemokines such as CXCL9, CXCL10, CXCL11, and CCL1 demonstrated variable expression levels in the TsCIA lungs with or without treatment. Besides CXCL10, it has also been suggested that other chemokines might be widely involved in the pathology of TsCIA-associated ILDs.

## Materials and Methods

### Ethics statement

All animal experiments were approved by the Medical Ethics Committee from the School of Medicine, Yunnan University, Yunnan Province, China. All experimental procedures were carried out according to the approved guidelines.

### Animals

Forty-eight adult male tree shrews (*Tupaia belangeri chinensis*, 150 g mean weight, 4–6-months old) were purchased from the Kunming Institute of Zoology (http://www.kiz.ac.cn/). Animals were routinely bred in an experimental animal room under the following conditions: 25 ± 2 °C temperature, 12 h/12 h light/dark cycle, and 50% relative humidity. Tree shrews were fed daily with 100 g of grain and 20 g of fruit. All animal studies and relevant protocols were reviewed and approved by the Ethics Committee from the School of Life Sciences of Yunnan University.

### Collagen-induced arthritis

Methods for the tree shrew collagen-induced arthritis (TsCIA) model were modified from the known rodent CIA protocol^45^. In brief, 2 mg/ml of bovine type II collagen (Sigma, USA) was prepared in 0.1 M acetic acid and emulsified in complete Freund’s adjuvant (Sigma, USA). Tree shrews were then subcutaneously injected with 0.25 ml of the above solution to induce experimental arthritis (20 tree shrews per group). The clinical arthritis index was scored as follows: level 4 = excess edema with joint rigidity; level 3 = pronounced edema with limited joint usage; level 2 = low to moderate edema; level 1 = slight swelling and/or erythema; and, level 0 = no swelling or erythema. Experimental tree shrews were weighed to monitor their health condition. *In vivo* drug treatments were conducted by single intraperitoneal (IP) injection of 1 mg/kg of CXCR3-specific antagonist NBI74330 (MedChem Express, USA).

### X-ray

X-rays were taken using a collimator type R-5 (Shimadzu, Japan) with a 30 mGy dose to assess skeletal deformities and bone erosion.

### Histology

All experimental tree shrews were anesthetized by subcutaneous injection of 10% chloral hydrate (400 μl per 100 g). Animals were perfused with 4% phosphate-buffered paraformaldehyde (PFA). Animal joints and lungs were removed, dissected, and sliced into 8-μm cross-sections. Sections were stained by hematoxylin and eosin (H&E), Sirius red, and solid green according to standard procedures. Masson staining was performed following standard protocols.

### Cell toxicity

Tree shrew peripheral blood mononuclear cells (PBMCs) were isolated with mouse Ficoll-Hypaque solution (Solarbio, China) by centrifugation at 1500 rpm for 20 min at room temperature, and then washed with PBS. Cells were counted using 0.05% trypan blue for subsequent experiments. The PBMCs (2 × 10^4^ cells per well) were seeded into 96-well plates supplied with complete RPMI 1640 medium and cultured at 37 °C with 5% CO_2_. Cell toxicity of NBI74330 was evaluated with MTT (3-(4,5-dimethyl-2-thiazolyl)-2,5-diphenyl-2H-tetrazolium bromide).

### Western blotting

Lung tissues from tree shrews were prepared with lysis buffer (Beyotime, China) containing protease inhibitors (Merck, USA). Rabbit anti-human polyclonal antibodies, including CCR3, CCR7, SLC2A4RG, TNFSF13B (BBI Life Science, China), and GAPDH (Beyotime, China), were used for Western blotting. Protein bands were visualized by chemiluminescence (Protein Simple, USA) and further analyzed by NIH ImageJ software.

### *In situ* hybridization (ISH)

All tree shrews were anesthetized and perfused with 4% PFA. Their lungs were removed, dissected, and sectioned into 30-μm cross-sections. To label both sense and antisense rRNA probes for ISH, tree shrew CXCL10 cDNA was subcloned into the pGEM T-vector (Promega, USA). rRNA probes for hybridization were prepared by DIG RNA Labeling Mix (Roche, USA). The ISH was performed per standard methods: Briefly, tissue sections were hybridized with rRNA probes at 60 °C overnight. Sections were incubated with anti-digoxigenin antibody conjugated with alkaline phosphatase (Roche, USA). The ISH signals were further developed with 75 μg/ml of nitroblue tetrazolium and 175 μg/ml of 5-bromo-4-chloro-3-indolyl phosphate substrate (Solarbio, China).

### Real-time PCR

Total RNA was extracted from fresh tree shrew lung tissue after digestion with TriZol (TaKaRa, Japan). The PCR primers were designed using Primer 5.0 (Table S1). The PCR cocktails were prepared using SYBR Premix (TaKaRa, Japan). The PCR program was: 1 cycle at 95 °C for 2 min, 40 cycles at 95 °C for 30 s, and 58 °C for 40 s. PCR was performed with an ABI 7300 Sequence Detection System (Applied Biosystems, USA). Data were analyzed by Sequence Detection Software (Version 1.2.2) provided by the manufacturer.

### *In vitro* transmigration

*In vitro* transmigration of tree shrew’s PBMCs was carried out using an AP48 48-well Boyden Chamber (Neuro Probe, USA) per the manufacturer’s instructions. Briefly, recombinant human CXCL10 (hCXCL10) protein (R&D Systems, USA) was added to the lower chamber. The Boyden chamber was separated by a 5 mm-diameter polycarbonate film with 5 μm-diameter pores. The PBMCs pre-treated with CXCR3-specific antagonist NBI74330 (MedChem Express, USA) were added into the top chamber. Cells on the membranes were counted after 24 h of culture in complete RPMI 1640 medium at 37 °C with 5% CO_2_.

### Statistics

All experiments were performed in duplicate and data were expressed as means ± SEM (standard error of mean). The statistical significance between two groups was analyzed using analysis of variance (ANOVA) followed by LSD tests. Values of *p* < 0.05 were considered statistically significant.

## Electronic supplementary material

Supplemental figures
