# Supplementary material for: Upregulation of chemokine CXCL10 enhances chronic pulmonary inflammation in tree shrew collagen-induced arthritis
Source: Sci Rep. 2018 Jul 3;8:9993. doi: 10.1038/s41598-018-28404-y (PMC6030082; doi:10.1038/s41598-018-28404-y)
Supplement: Supplementary file 1 — Supplemental figures [file 41598_2018_28404_MOESM1_ESM.pdf]

Upregulation of chemokine CXCL10 enhances chronic pulmonary inflammation in tree shrew collagen-induced arthritis

Bo GAO[1,2*], Jie LIN[2,3*], Zongmin JIANG[1,2], Zhongshan YANG[4], Haijing YU[1,2], Lei DING[1,2], Min YU[1,2], Qinhua CUI[1,2], Neil DUNAVIN[5], Ming ZHANG[6#], Meizhang LI[1,2#]

[1]Lab of Biochemistry & Molecular Biology, School of Life Sciences, Yunnan University, Kunming, Yunnan 650091 CHINA

[2]Key Lab of Molecular Cancer Biology, Yunnan Education Department, Kunming, Yunnan 650091 CHINA

[3]Lab of Monoclonal Antibody Engineering &Technology, School of Life Sciences, Yunnan University, Kunming, Yunnan 650091 CHINA

[4]Lab of Pathogen Biology and Immunology, Yunnan Traditional Chinese Medicine University, Kunming, Yunnan 650500 CHINA

[5]Division of Hematologic Malignancies and Cellular Therapeutics, Kansas University Medical Center, KS 66205 USA

[6]Yunnan Key Laboratory of Stem Cell and Regenerative Medicine, Institute of Molecular and Clinical Medicine, Kunming Medical University, Kunming, Yunnan 650500 CHINA

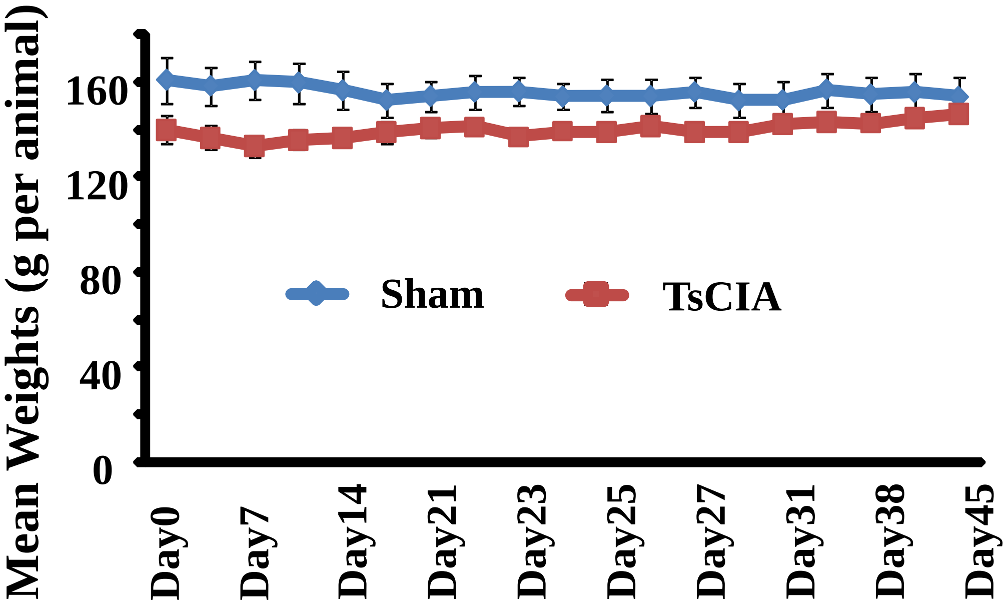

Figure S1. Average body weights between the TsCIA and control tree shrews.

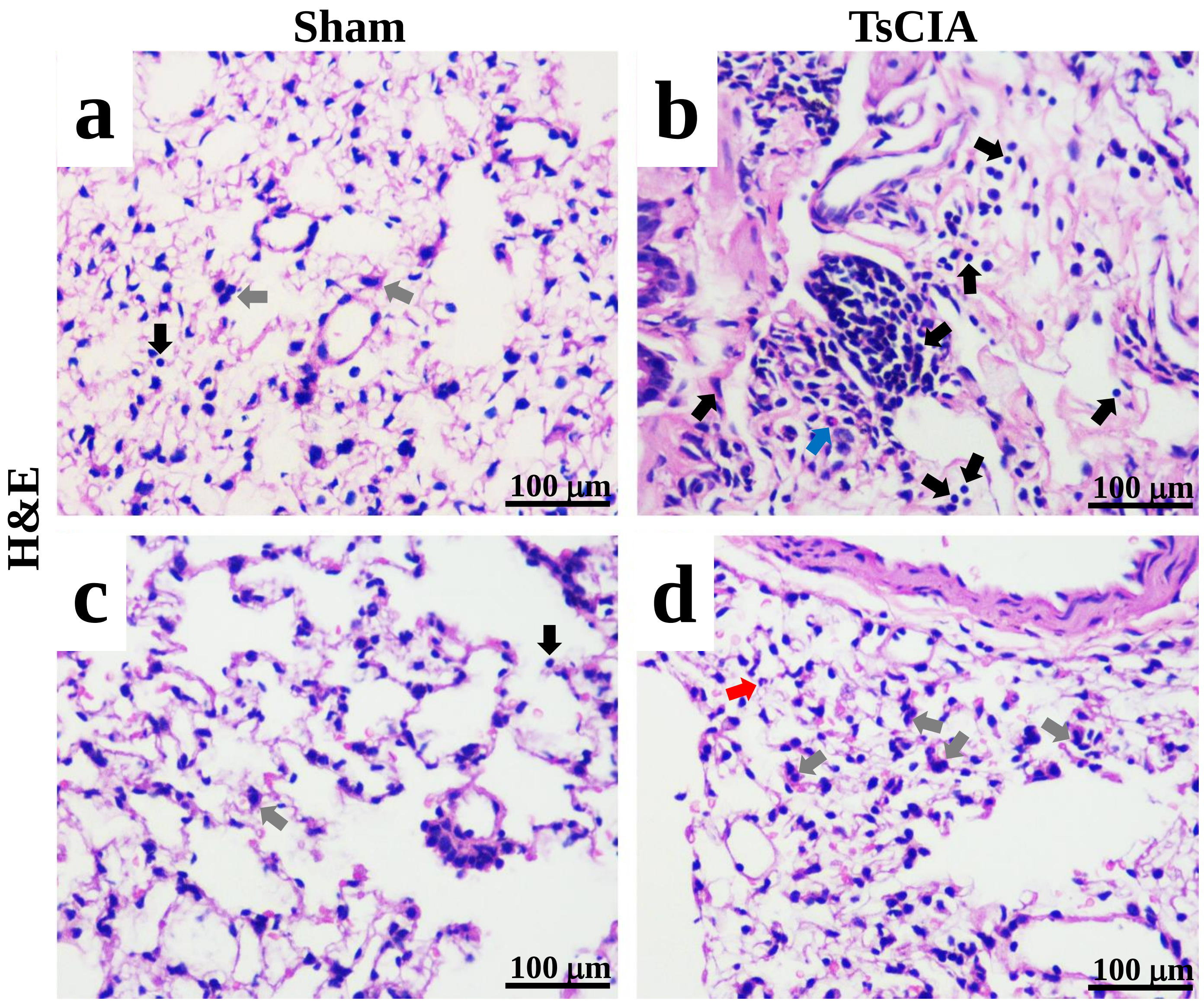

Figure S2. Infiltration of different types of inflammatory in the lungs of TsCIA. Identification of inflammatory cell types between the control (**a** and **c**) and TsCIA (**b** and **d**). Black arrows indicate lymphocytes, gray arrows indicate monocytes, Blue arrow indicate eosinophils and Red arrow indicate neutrophils. Scale bar, 100µm.

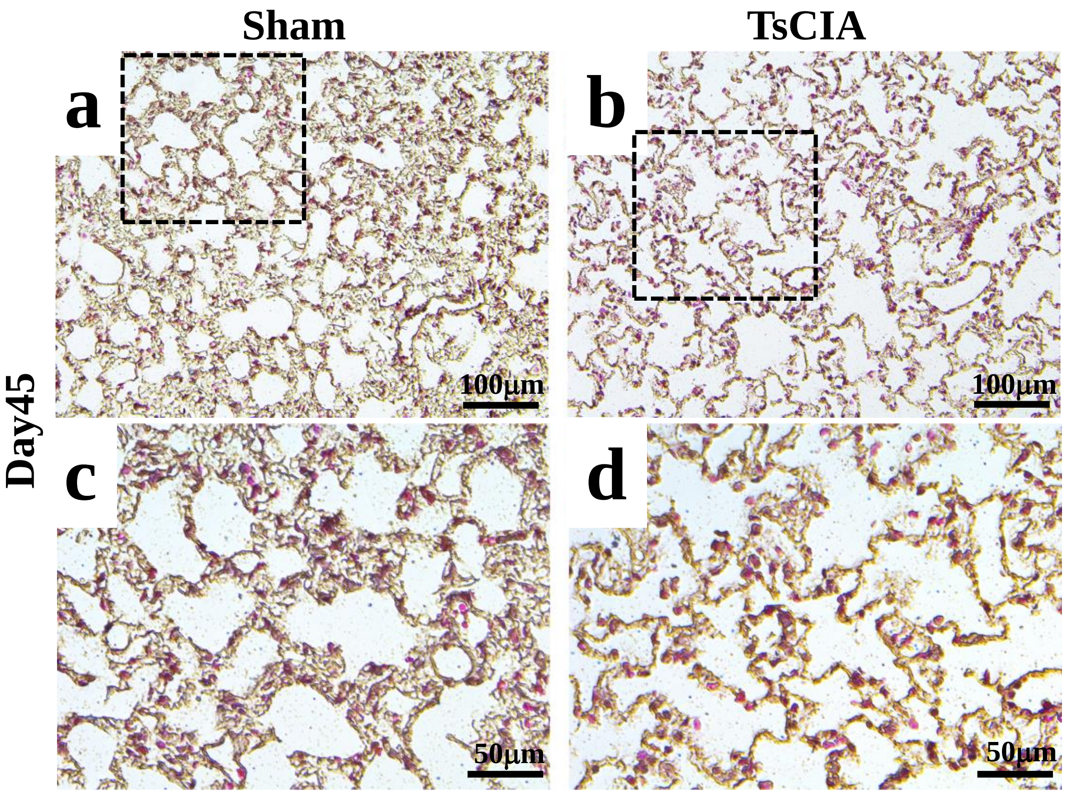

Figure S3. **Pulmonary fibrosis was not detected in TsCIA**. (**a-d**) Representative Masson staining of lung cross sections from the control (**a** and **c**) and TsCIA (**b** and **d**) groups. Scale bar:100μm (**a** and **b**); 50μm (**c** and **d**).

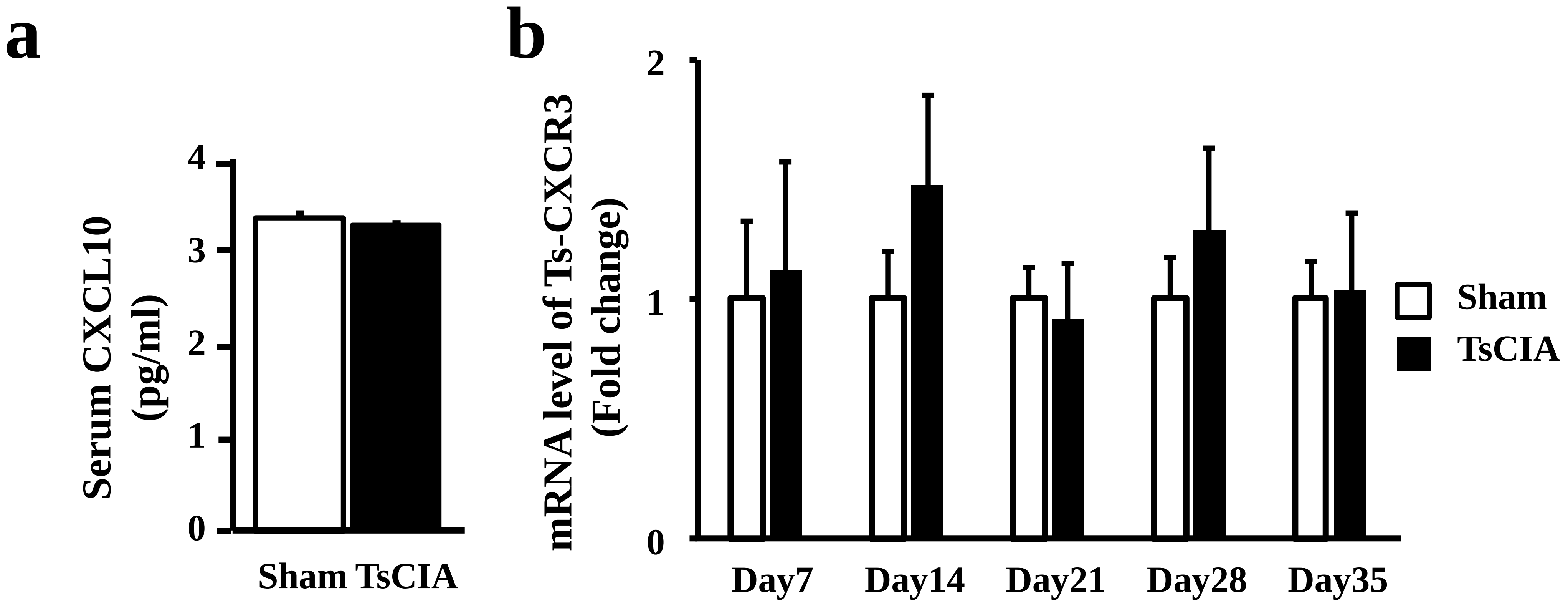

Figure S4. (**a**) The protein level of CXCL10 detected in peripheral blood serum. n=3 wells per group. (**b**) Quantitative analysis of mRNA levels of CXCR3 in PBMCs. n=3 wells per group. Error bars indicate SEM.

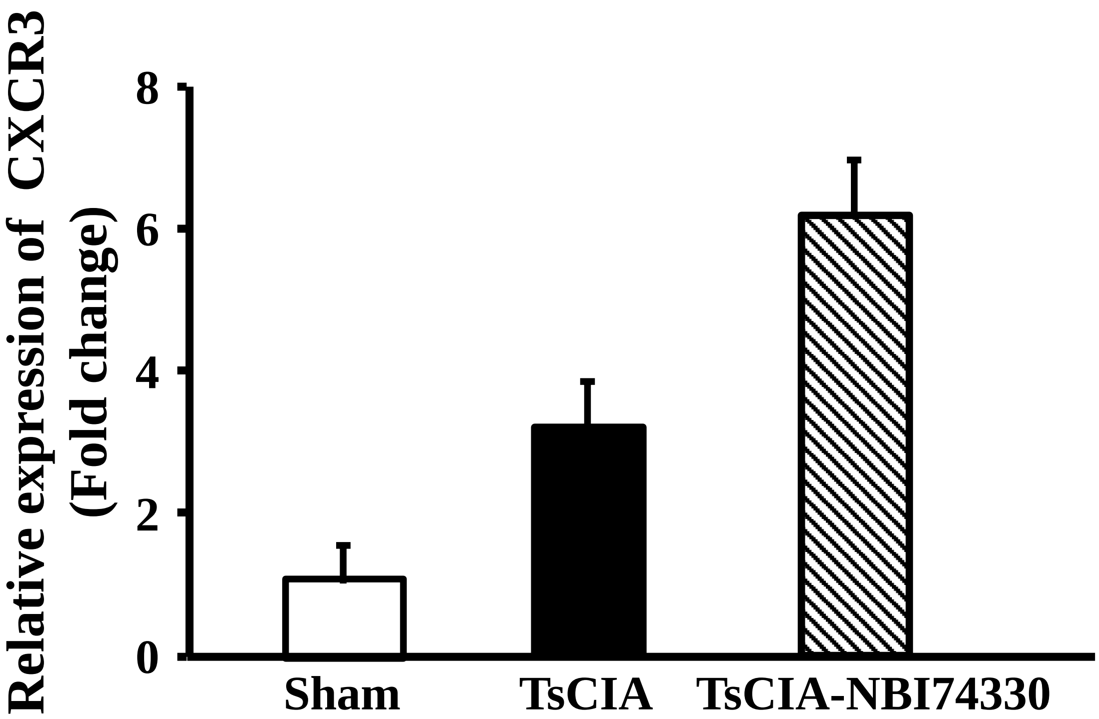

Figure S5. The mRNA level of CXCR3 was detected in lungs. n=3 wells per group. Error bars indicate SEM.

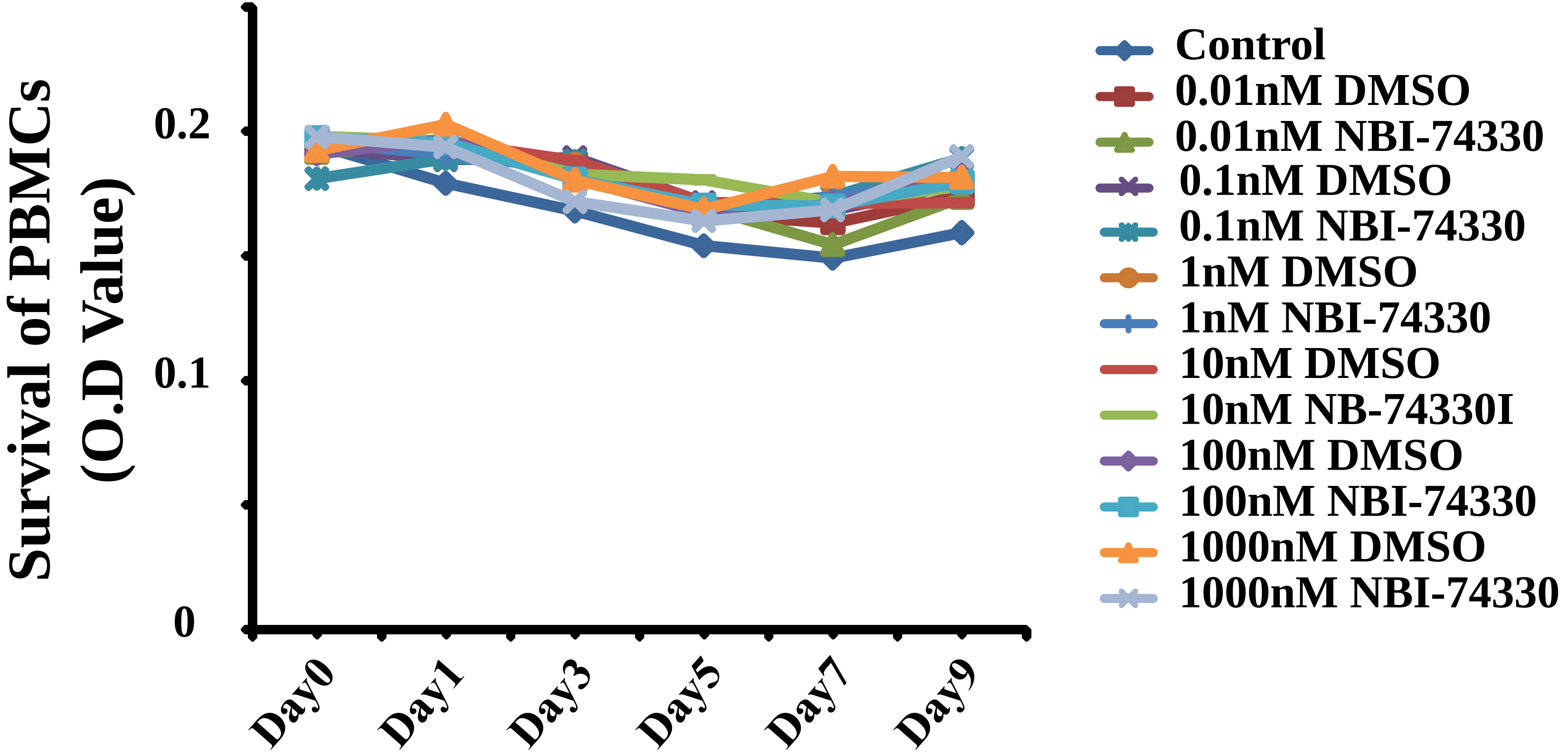

Figure S6. Survival of tree shrew PBMCs after treatment with NBI74330. Survival of tree shrew PBMCs was quantified after treatment with 0.01–100nM NBI74330 (n=3 wells per group. Error bars indicate SEM).

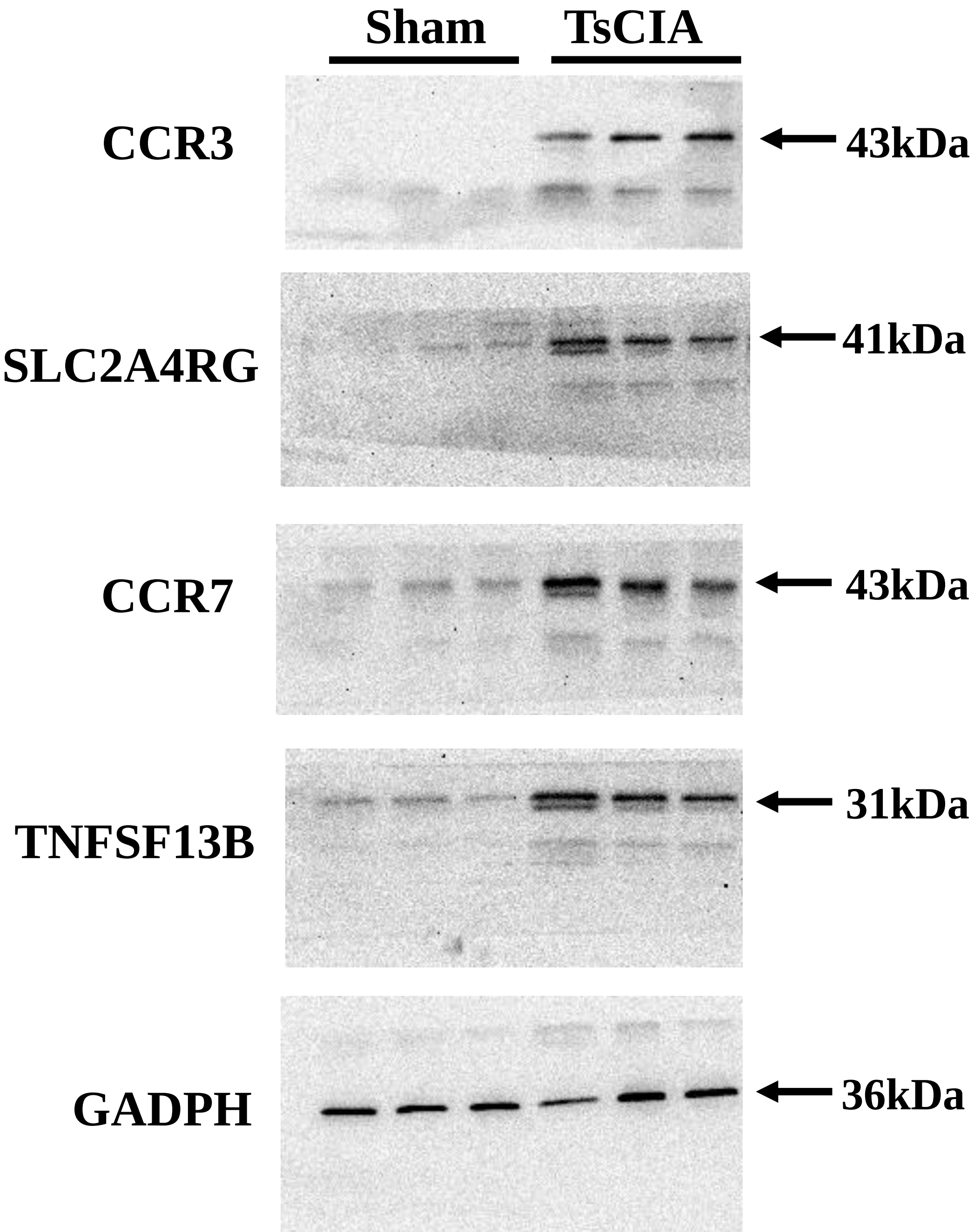

Figure S7. The original exposure blot images for supporting figure 2G. The band size of CCR3, SLC2A4RG, CCR7, TNFSF13B and GADPH are 43kDa, 41kDa, 43kDa, 31kDa, 36kDa, respectively.

Supplemental table 1.

Primer sequences for Realtime PCR.

Name	Sequence
18S forward	5'-CAGCCACCCGAGATTGAGCA -3'
18S reverse	5'-TAGTAGCGACGGGCGGTGTG -3'
CXCL10 forward	5'- GGAGTGCCTCTCTCTCGAAC -3'
CXCL10 reverse	5'- CGTGGACAAGATTGACTTGC -3'
CXCR3 forward	5'- GCTTGTGGACACTCTCATGG -3'
CXCR3 reverse	5'- TTGACTCCCACAAAGGCATA -3'
TNFα forward	5'- CGGCTCACGTTATAGCAAAC -3
TNFα reverse	5'- TGGTTGTCTACCAGCTGCAT -3
TGFβ2 forward	5'- CTGCTAATGTTATTGCCCTCCTA -3
TGFβ2 reverse	5'- TTCATGTATCCATTTCCACCC -3
VEGF forward	5'- CTACCTCCACCATGCCAAGT -3'
VEGF reverse	5'- CAAGCCCTCGTCATTACAGC -3'
PAD 4 forward	5'- CAAAGTGAAGCCGACCAGAG -3'
PAD4 reverse	5'- CCTTGTCATCTTCGCAGTCC-3'
MMP13 forward	5'- CCTCTGAATTTCACCCGACT -3'
MMP13 reverse	5'- CAGAAGACCAGAGGGTCCAT -3'
IL1β forward	5'- CAAGACAATAAGCCCACCCT -3'
IL1β reverse	5'- GCTTTGTCCCGATTTCTGTT -3'
IL2 forward	5'- CTTGTCTTGCATTGCCTTCA -3'
IL2 reverse	5'- TGTGGCCTTTCTGGGTGTAT -3'
IL6 forward	5'- GCTTGATGGTGAAGATGCAG- 3
IL6 reverse	5'- CCGAGAAGCCCTCACAGTAT -3
IL8 forward	5'- GCTGGCTATTGCTCTGTTGG -3'
IL8 reverse	5'- GCATTGGCATCGAAGTTCTG -3
CXCL9 forward	5'- GACGCTGTGTCTGCATTGA -3
CXCL9 reverse	5'- TTTCTCACAGAAAGGGCTTG
CXCL11 forward	5'- AAGGAGGACGTTGTCTTTGC -3'
CXCL11 reverse	5'- TTGCTTGCTTCGAGTTAGGA -3'
CXCL13 forward	5'- GTCTCCAGTGCATGGTGTTC -3'
CXCL13 reverse	5'- TTCAATTTGGCGTATCTGGA -3'
CCL1 forward	5'- TCCAACTGCTGCTTCTCATT -3'
CCL1 reverse	5'- TTTACAGAGCGGTAGGAGCA -3'

CXCR1 forward	5'- CGTCTACGCCCTGGTGTTC -3'
CXCR1 reverse	5'- AAATCCAGCCCTTCTCCCT -3'
CXCR2 forward	5'- CGGGTCATCTTTGCTGTTGT -3'
CXCR2 reverse	5'- GGCATCTAAAGCGTGGTCAA -3'
CXCR4 forward	5'- AATCTTCCTGCCCACCATCT -3'
CXCR4 reverse	5'- GGTGCAGCCTGTACTTGTCC -3'
